# Pediatric Dental Education in China: Current Status, Challenges, and Pathways to Improvement—A Narrative Review

**DOI:** 10.3390/dj14070456

**Published:** 2026-07-20

**Authors:** Yandi Chen, Yan Wang, Rui Shu, Mingmei Meng, Jing Zou, Qiong Zhang

**Affiliations:** 1 State Key Laboratory of Oral Diseases & National Center for Stomatology & National Clinical Research Center for Oral Diseases, National Research Center for Educational Materials, Department of Pediatric Dentistry, West China Hospital of Stomatology, Sichuan University, Chengdu 610041, China; chenyandi1992@scu.edu.cn (Y.C.); wangyan1458@scu.edu.cn (Y.W.); shurui@scu.edu.cn (R.S.); mengmm@scu.edu.cn (M.M.); zoujing@scu.edu.cn (J.Z.); 2 Department of West Outpatient, West China Hospital of Stomatology, Sichuan University, Chengdu 610031, China

**Keywords:** pediatric dentistry, China, children’s oral health, dental education, health policy

## Abstract

Pediatric dental diseases, notably dental caries, malocclusion, and dental trauma, remain a significant public health challenge in China, exerting considerable impact on children’s oral and general health. Despite growing societal demand for pediatric dental care, China faces a critical shortage and uneven geographic distribution of pediatric dentists, alongside persistent urban–rural disparities in access to oral health services. These challenges underscore the urgent need for educational reforms, workforce policy adjustments, and assessment of the strengths and limitations of recent training initiatives to enhance pediatric dental service delivery. This narrative review aims to: (I) describe the current structure of pediatric dental education in China and recent reforms, notably the integration of degree education with standardized residency training; (II) summarize the epidemiological burden of dental diseases among Chinese children, together with contributing social and geographic factors; (III) outline national policy initiatives targeting children’s oral health and existing gaps in implementation; and (IV) discuss innovations in dental education, including student-centered pedagogies and humanities integration. This review provides a comprehensive overview of pediatric dentistry in China and offers strategic recommendations to strengthen workforce training, expand service access, and reduce the burden of pediatric oral diseases.

## 1. Background

Childhood represents a critical window for dental development, underscoring the necessity of specialized pediatric dental care. Over the past decade, pediatric dentistry in China has gained increasing recognition as a distinct discipline dedicated to children across various age groups [1]. Nevertheless, oral diseases, particularly dental caries, remain a formidable public health concern. Untreated early childhood caries (ECC) exerts detrimental effects on children’s overall health and development [2]. The prevalence of malocclusion and dental trauma further compounds this burden, necessitating treatment approaches that not only address dental issues but also integrate psychological and social dimensions of children’s oral health and well-being [3].

Recognizing the importance of pediatric oral health, an increasing number of dental schools have initiated specialized pediatric dental programs [1]. The National Call to Action to Promote Oral Health underscores the need for a more diversified and adequately staffed dental workforce to address the oral health needs of children [4]. In 2021, China reported approximately 278,000 dentists, with an annual influx of 20,000 new practitioners, yielding a dentist-to-population ratio of 1:5000 that aligns with WHO recommendations [5,6]. Yet this aggregate figure masks profound disparities: the Pediatric Dentistry Committee of the Chinese Stomatological Association comprises merely 4475 members, serving a child population of 256.15 million [7], reflecting severe workforce shortages. Moreover, disparities in access to oral health resources persist between urban and rural areas, with urban residents generally benefiting from superior oral health services [8,9,10]. Integrating oral health initiatives into poverty alleviation programs could help bridge these gaps and ensure equitable access to dental care nationwide [11], while dental insurance plays a crucial role in enhancing service utilization, though financial barriers remain significant [12]. Notably, China’s dental education system has undergone structural reforms, with ongoing efforts to integrate standardized residency training with postgraduate programs, yet the clinical impact of these reforms remains insufficiently documented.

Previous studies have largely focused on individual dimensions of pediatric oral health in China, such as disease burden, preventive care and early intervention strategies, and geographic and socioeconomic barriers to care, without integrating these into a comprehensive analysis of education, policy, and practice. This review addresses these gaps by providing a holistic examination encompassing: (I) the structure and recent reforms of training programs; (II) the burden of major pediatric dental diseases and associated social and geographic determinants; (III) national policy frameworks and existing gaps in implementation; and (IV) innovations in education, including student-centered pedagogies and humanities integration. By synthesizing evidence across these domains, we aim to identify actionable strategies for strengthening pediatric dental workforce capacity and improving children’s oral health outcomes nationwide.

## 2. Methods

A comprehensive search was conducted across PubMed/MEDLINE, Embase, Web of Science, Scopus, and China National Knowledge Infrastructure (CNKI), supplemented by policy documents from the State Council of the People’s Republic of China, the National Health Commission of the People’s Republic of China, the China CDC, and the China Oral Health Foundation. Search terms centered on pediatric dental education in China (e.g., “pediatric dentistry”, “pedodontics”, and “dental education”), supplemented by terms related to dental diseases (e.g., “dental caries”, “malocclusion”, and “dental trauma”) and health policy (e.g., “dental hygienist”, “hierarchical diagnosis and treatment”, and “social insurance”).

Two reviewers independently screened titles and abstracts, with full-text assessment of potentially eligible studies. Disagreements were resolved through discussion or consultation with a third reviewer. Although narrative in design, this review incorporated systematic elements to enhance transparency and minimize selection bias.

## 3. Overview of Pediatric Dentistry Education in China

As of March 2021, available data indicated that a total of 32 colleges and universities across China have established dedicated teaching and research departments for pediatric dentistry and offer professional courses [1]. China has established a comprehensive medical education system that comprises medical schools, postgraduate medical education, and continuing education. The primary component of this system is the “5 + 3” model, which consists of a 5-year undergraduate program in clinical medicine, followed by a 3-year residency training program or a postgraduate program [13]. The professional degree training in stomatology follows a progressive, continuous training system that begins with a bachelor’s degree, advances to a professional master’s degree, and ultimately leads to a professional doctorate. Continuing medical education is pivotal for healthcare professionals to acquire and apply new knowledge, theories, and skills following the completion of medical school education and standardized training.

In China’s undergraduate dental education, there is a greater emphasis on basic and clinical medicine courses compared to the United States and Europe. Course hours allocated to these courses constitute a larger proportion of the undergraduate curriculum, thereby limiting the instructional hours and training intensity dedicated to dental professional programs [14]. Similar to its western counterpart, pediatric dentistry in China is typically integrated into master’s degree education or offered as part of continuing education programs, highlighting its role as a specialized field within dentistry. The curriculum for pediatric dental education in China encompasses a wide range of subjects, including child behavior management, growth and development, preventive dentistry, dental trauma management, and restorative dentistry for children. Students also acquire knowledge in the diagnosis, treatment, and management of various oral diseases, thereby enhancing their understanding of pediatric dentistry and preparing them to address clinical complexities in their future practice.

In recent years, there has been a growing emphasis on incorporating research and evidence-based practice into pediatric dental education. Dental schools encourage students to engage in research projects related to pediatric dentistry, in order to expand their knowledge in this field. Furthermore, to enhance students’ practical skills, dental schools frequently provide opportunities for them to gain hands-on experience through clinics and hospital rotations. These experiences enable students to acquire proficiency in common pediatric dental procedures, such as dental examinations, cleanings, restorative treatments, and extractions, under the guidance of experienced pediatric dentists. This supervised clinical practice not only reinforces theoretical knowledge, but also equips students with the confidence and competence required in their future professional practice.

Presently, the evaluation of dental students in China primarily emphasizes dissertation research and clinical proficiency. Within this context, a “dual track integration” approach has recently been implemented, merging master’s degree education in clinical medicine with standardized residency programs to enhance the alignment between academic credentials and clinical competence. An assessment at a national clinical research center for oral diseases evaluated this integrated model through examinations and faculty-trainee surveys [15]. Results indicated that integrated-track trainees achieved significantly higher scores in professional knowledge and clinical specialty skills, while conventional trainees performed stronger in public health skills. Faculty further noted greater learning motivation among integrated-track trainees, but higher operational standardization among conventional trainees [15]. These findings suggest that the integrated model effectively consolidates academic and clinical training, though continued optimization is needed. This innovative training model underscores the critical requirement for systematic and standardized clinical competence training, emphasizing the development of core clinical practice skills and aiming to enhance students’ comprehensive clinical capabilities and work competence [15].

Overall, pediatric dental education in China has made significant progress, with ongoing efforts dedicated to improving the educational and training quality in this specialized domain.

## 4. Issues and Challenges

### 4.1. Dental Diseases in Children and Social Burden

Pediatric dental health is a pressing global public health concern, with significant social and financial burdens [16,17]. Dental caries, malocclusion and dental trauma are significant issues, with prevalence rates rising and impacting children’s dental function, facial aesthetics, and psychological well-being. Addressing these challenges requires comprehensive preventive strategies, improved access to care, and greater emphasis on pediatric dental health in public health initiatives.

#### 4.1.1. Dental Caries

Despite being preventable, dental caries, particularly early childhood caries (ECC), remains highly prevalent, affecting approximately 560 million children worldwide and ranking as the 12th most common disease in the 2015 Global Burden of Disease Study [18,19]. In China, the fourth national oral health survey (2015–2017) revealed alarming statistics, with 70.9% of 5-year-old children suffering from dental caries, but only 4.1% of decayed teeth received treatment, far below the WHO’s recommended 40–60% for middle-income countries [20]. Implementing preventive and control strategies is essential to address dental caries in children. Advocacy charities, such as the Alliance for a Cavity-Free Future, have been established seeking to confront and reduce the disease burden of caries through prevention [21]. However, significant gaps remain in caries prevention and treatment globally.

#### 4.1.2. Malocclusion

Malocclusion, characterized by irregular teeth positioning or misalignment of dental arches, is recognized as one of the three major oral diseases by the World Health Organization. Various factors, such as dental caries, traumatic dental injuries, hereditary factors, and environmental factors, contribute closely to the development of malocclusion [22]. In children, malocclusion can adversely affect dental and maxillofacial function, mastication, facial appearance, general psychological health, and social behaviors [23].

The prevalence of malocclusion has observed a significant increase, rising from 40% in the 1960s to 67.82% in the 2000s [24]. Studies on Chinese children aged 2–7 years indicated a pooled prevalence of malocclusion at 45.50%, based on a literature review spanning from 1988 to 2017 [25]. Another review by Min Lin et al. reported a pooled national prevalence of malocclusion among Chinese schoolchildren at 47.92%, based on literature spanning from 1991 to 2018 [26]. Regional disparities in socioeconomic status, dietary habits, and cultural contexts may contribute to variations in the prevalence of malocclusion across different regions of China [27]. Due to its impact, malocclusion has become a significant and pressing issue necessitating collaboration among pediatric dentists, epidemiologists, and government entities.

#### 4.1.3. Dental Trauma

Dental trauma commonly results from falls in preschool students, likely due to their immature neuromotor systems, rendering them more susceptible to such injuries compared to adults [28]. Among teeth, the upper central incisors are especially vulnerable to trauma due to their prominent position [29]. It is important to note that dental trauma can negatively impact the development of permanent teeth and result in various developmental disorders [30].

The prevalence of dental trauma is 25% among school children and 33% among adults who have experienced trauma to their permanent dentition. Most of these injuries occur before the age of 19 [31]. Dental trauma accounts for a significant proportion of emergency visits, ranging from 4.35% in Beijing to 5.18% in Shanghai [32,33,34]. Globally, the prevalence of dental trauma varies, with approximately one-third of preschoolers and one-quarter of adolescents and adults experiencing dental trauma at least once in their lifetime [35]. The time taken for the treatment of dental trauma significantly affects the prognosis of the affected teeth. Challenges arise from the lack of standardized treatment for traumatic injuries to deciduous teeth and the varying levels of dental care available in China. Standardizing the management of traumatic dentition is crucial due to the pivotal role of emergency treatment in determining prognosis [36]. A study found that one-quarter of individuals with permanent tooth fractures express dissatisfaction with the aesthetic appearance of the restoration [37].

### 4.2. Deficiencies in Dental Education

There exists an imbalance between clinical training and academic research in the Doctor of Medicine program in China, highlighting the need to address concerns surrounding clinical training. Academic research and output often take precedence over the development of clinical teaching competency. The increased focus on academic research requirements can lead to a reduction in the time available for clinical training. All respondents acknowledged working outside of regular hours and reported insufficient sleep due to their current working patterns. Furthermore, there are shortcomings in the oversight of standardized training [38]. Empirical evaluations of the integrated residency-master’s training model have corroborated these concerns, noting that trainees in the integrated track face competing demands between degree coursework and clinical rotations, with theoretical study time compressed and teaching quality affected in some institutions [15]. Faculty surveys further indicate that mentoring concepts for integrated-track trainees remain inconsistent, and that training content and processes require clearer standardization to ensure equitable outcomes across diverse training sites [15].

In addition to acquiring fundamental dental knowledge through traditional lecture-based education, it is crucial for dental students to develop interpersonal skills, independent thinking abilities for disease diagnosis and treatment, and most importantly, critical thinking skills for lifelong learning [39]. The current rigid medical training mode calls for reform. The student-centered educational approach, which emphasizes skills development, is not adequately implemented, with many courses prioritizing medical technology and clinical training. The conventional teaching method, which revolves around teacher-centered, class-oriented didactic lectures and an examination-driven curriculum, fails to address the development of the students’ interests and autonomy [40].

Expertise in dentistry education is important, but so are humanistic qualities. A pediatrician’s clinical experience and humanistic spirit have a positive impact on behavior guidance for children and contribute to fostering a good relationship between children and dentists [41]. Medical humanities education should be emphasized to instill social responsibility, compassion, and empathy in medical students. This education also helps dental students build personal and professional values on a strong foundation [42]. However, the lack of sufficient medical humanities education and faculty expertise in this area remains a significant issue [43].

### 4.3. Urban–Rural Disparities and Oral Diseases

Rural areas in China continue to face pronounced challenges in pediatric oral health. The fourth national oral health survey revealed marked geographic gradients in disease burden and service access: among 5-year-old children, the caries prevalence was 65% in urban areas compared with 76% in rural areas; among 12-year-old children, the prevalence of permanent tooth caries was 28% in urban areas compared with 42% in rural areas [20]. Rural children also demonstrated lower frequencies of tooth brushing and reduced use of fluoride toothpaste, while the density of dental professionals and oral health facilities in rural regions remained substantially below urban levels [20]. Negative attitudes and improper behaviors towards oral health, particularly among non-parental caregivers, the lack of concern for oral diseases, low family income, and a shortage of specialist dentists are all risk factors for dental caries, resulting in lower levels of oral health in rural children compared to urban areas [44]. Untreated dental issues in rural children can lead to premature tooth loss. Another group that requires attention is rural-urban migrant children, considering their disadvantaged family economic situation, lack of parental attention, and limited access to basic healthcare.

Inadequate access to oral health care poses a significant threat to the oral health of the rural population. Poverty and poor living conditions in rural areas discourage dentists from practicing there. Conversely, there is an oversupply of dentists in urban areas, making it difficult for newly graduated oral medical students to find employment in these locations, leading to a shortage of dentists in rural areas. Additionally, dentists in rural areas have lower levels of education and less access to professional training [45].

## 5. Solutions

### 5.1. Implementation of the Policy

#### 5.1.1. Establish an Efficient Dental Hygienist System

The “Healthy China 2030 [46]” blueprint emphasizes the importance of prioritizing people’s health, with a particular focus on healthcare at the grassroots level and preventive measures. The government plays a crucial role in preventing and controlling oral diseases. To achieve this, it is necessary to establish national and regional oral health centers and develop networks dedicated to oral disease prevention and control. These measures will provide the necessary foundation for comprehensive oral health management throughout an individual’s lifespan. As shown in Table 1, the Chinese government has taken steps to tackle oral health issues.

These initiatives reflect a strategic shift toward preventive-oriented pediatric oral health care in China. Building on this strategic shift, realizing these ambitions will require not only sustained fiscal commitment and workforce expansion but also the establishment of clearly defined professional roles within the oral health team. International experience suggests that dental hygienists serve as critical links between policy intent and community-level delivery, bridging gaps in prevention, education, and care coordination.

Different countries follow distinct paths and practices in promoting dental hygienists, yet the beneficial effects are universally evident, as shown in Table 2. The presence of dental hygienists enhances oral health standards, plays a critical role in prevention and education, reduces the incidence of oral diseases, and alleviates the burden on the healthcare system. By promoting and supporting the work of dental hygienists, countries have achieved significant public health benefits.

The Chinese Stomatological Association is actively working to establish an official hygienist system in China. On 24 May 2024, the Ministry of Human Resources and Social Security of China announced 19 new professions, including dental hygienists in the medical and health sector. Dental hygienists are defined as professionals engaged in oral health services, including disease prevention, healthcare, and assistance in diagnosis and treatment. Well-educated dental hygienists, dentists, and pediatric primary care physicians can play a vital role in promoting dental knowledge and improving the oral health of Chinese newborns and children [58]. However, there is still considerable progress needed in enhancing public oral education and children’s oral health care due to the historical neglect of oral health issues. The future will witness a growing demand for dental practitioners who prioritize preventive-oriented oral healthcare. Therefore, it is essential to educate dental students on preventive dentistry and dental hygiene, enabling them to impart the concept of preventive-oriented dental care to parents or caregivers in the future.

#### 5.1.2. Hierarchical Diagnosis and Treatment

The overall supply of oral healthcare services in China is insufficient and unevenly distributed, resulting in a lack of tailored oral health services. Thus, it is essential for dentists to implement accurate diagnoses, appropriate treatments, and preventive measures. To drive innovation and development in oral health services, accelerating the construction of a grassroots network for the prevention and control of oral diseases is crucial. This can be achieved by enhancing the training of grassroots oral health service coordinators and increasing the capacity of grassroots health service organizations in oral disease prevention and control. Moreover, planning and coordinating social resources can contribute to the overall improvement of oral health services.

Children’s oral care is significantly influenced by the family environment. Tooth brushing is a critical oral health measure and the most common, cost-effective form of home care for mechanically removing plaque and soft deposits [59]. Contracting pediatric oral healthcare services can provide children with comprehensive, continuous, and coordinated basic oral health services. This can be achieved by engaging families through contractual services between family doctors and parents. When parents receive professional oral health guidance, they better understand the significance of children’s oral healthcare and are more likely to accept contracted pediatric oral health services. This long-term and stable contractual service relationship can expand the scope of children’s oral health services and provide comprehensive and ongoing intervention for oral diseases in children.

Another model for pediatric dental healthcare is the collaboration between family doctors and community-based dental care services. This model ensures consistent follow-up visits due to their proximity to children’s families. Long-term and stable communication between doctors and parents allows for personalized oral hygiene advice for children, as well as early diagnosis and prevention of oral diseases. Collaboration with caregivers also improves oral health behaviors and awareness among parents, ultimately enhancing children’s oral healthcare [60,61].

#### 5.1.3. Poverty Alleviation via Health Care

To address the oral health issues in rural children, the government could allocate funds for oral health education programs specifically targeted at this population [62]. The China Oral Health Foundation launched a public welfare fundraising campaign, the Mountain Daisy Dream Building Project, to promote children’s oral health education in rural areas, talent training, and other public welfare poverty alleviation work [63]. Initiated in 2013, the “People’s Health Service Action” focuses on delivering primary medical services and enhancing primary healthcare capabilities. Annually, medical and health institutions nationwide are organized to participate, with oral health being a major component. Activities include specialized oral health examinations, caries prevention and treatment, and oral hygiene education.

Ineffective communication between migrant children and their busy working parents also contributes to poor oral health outcomes. To improve their oral health, both children and caregivers should actively participate in initiatives aimed at enhancing oral health knowledge and promoting healthy behaviors. It is imperative to raise social awareness about the oral health needs of migrant children and implement relevant policies. Future educational programs should be tailored to migrant populations, considering varying levels of oral health knowledge, behaviors, and parental practices [64].

To meet the oral healthcare needs of the entire country, China’s dental workforce must continue to strengthen professional education and clinical competencies. Building on existing policy frameworks, it is recommended that incentive mechanisms be further refined to encourage dental university graduates to serve in less developed regions, thereby promoting balanced regional development of dentistry and equitable access to high-quality oral healthcare. The government can also learn from the experiences of other countries by focusing on training dental assistants and emphasizing preventive oral healthcare [65].

### 5.2. Increase in Social Insurance Coverage

Research indicates that the average dental expenditure per capita in China is approximately $20.55, with over 90% of oral healthcare costs paid out-of-pocket [66]. Currently, treatment fees for oral diseases are typically not covered by basic medical insurance plans in most Chinese cities. In the future, it is recommended to provide basic oral health care and management services focusing on prevention to children and adolescents, aiming to reduce the incidence of oral diseases at their source. Children with ECC can also benefit from insurance reforms covering preventive oral care services [67].

To establish a comprehensive oral health insurance support system, the government may prioritize basic health insurance as primary coverage, with commercial health insurance as a supplementary option. Encouraging the expansion of commercial insurance coverage for oral diseases can enhance the coverage of high-value oral medical services [68].

### 5.3. Innovations and Development in Pediatric Dental Education

#### 5.3.1. Pediatric Dentistry in Undergraduate and Continuing Medical Education

Currently, the dentistry training program in China lacks emphasis on clinical practice requirements for undergraduate students in pediatric dentistry. This gap exists between undergraduate programs and the specialized knowledge and skills required for pediatric dental experts. After graduation, many undergraduate students struggle with providing independent clinical care in pediatric dentistry. This is due to difficulties in developing clinical skills within a limited timeframe and effectively applying classroom theory to clinical practice.

To address this issue, undergraduate intern programs can help students develop clinical thinking, operational abilities, and consolidate theoretical knowledge in pediatric dentistry. Pediatric dental expertise primarily derives from post-graduate training and continuing medical education. However, the implementation of continuing medical education varies across regions, particularly in economically underdeveloped areas, posing challenges to ensuring its quality [69].

#### 5.3.2. Innovations in Training Model

China has been continuously exploring the reform of the training mode in medical education by optimizing educational objectives, integrating teaching contents, enriching teaching modes, improving the education system, and innovating the evaluation and assessment system. Special attention has been given to cultivating professionalism and innovation.

The new teaching models promote student engagement, autonomy, and overall competence. Problem-Based Learning (PBL) is an increasingly popular student-centered and inquiry-based group learning approach [70]. It has been widely embraced by many dental and medical schools in China and has been shown to be more effective than traditional teaching methods [71,72]. However, traditional PBL faces limitations in terms of scheduling regular meetings and implementing it across different geographical and clinical departments. Online PBL through platforms like WeChat has emerged as a solution to overcome these limitations, allowing for successful and promising teaching methods for dental internships [73]. Additionally, Case-Based Learning (CBL) has been found to be superior to lecture-based teaching techniques and can enhance the general competency of dental students [74]. Apart from PBL and CBL, other innovative teaching methods such as mobile learning, blended offline and online teaching, the flipped classroom, and online learning have been adopted to improve teaching quality and meet the needs of students [75]. Integrating online learning into dental education can help address the shortage of teaching resources, facilitate remote learning, increase student engagement and cultivate positive attitudes [76].

In addition to technical skills, humanistic practice ability should be integrated into the entire process of talent cultivation in higher education institutions. Rather than being parallel and separate from clinical learning, the humanities need to be integrated with the clinical components of dental education. Medical humanities education should be provided during education to promote the integration of humanities education and professional education. Mastery of humanistic practice ability helps build dentist-patient relationships, which, in turn, leads to the provision of high-quality and appropriate healthcare, as well as patient safety and wellbeing [77,78].

Improvements in standardized residency training institutions can help achieve high-quality education [79]. The physician training process in China emphasizes the development of values and work ethic. For graduate dentists, there may be issues with building trust and establishing good doctor–patient relationships with anxious patients. Undergraduate students who are new to providing dental therapy in similar situations may be more prone to feeling unconfident due to a lack of clinical experience. Adequate technical training should be provided to facilitate building doctor–patient relationships before beginning practice with actual patients. Students should be encouraged to communicate and focus on patient feedback, as these experiences are important for improving empathy [80].

Innovative teaching methods may focus on effective communication and behavioral management strategies for working with young patients who may feel anxious or uncooperative during dental visits. A deeper understanding of child psychology and behavior may be integrated into pediatric dental education to help dentists build rapport with young patients and ease their anxiety.

Emerging digital technologies, including artificial intelligence (AI), are beginning to complement pediatric dental education. Deep learning models have demonstrated proof-of-concept feasibility in detecting extensive dental caries from intraoral scans in children, with moderate sensitivity and comparable diagnostic agreement to practitioner assessments in exploratory studies [81]. Systematic reviews further indicate that AI applications in pediatric dentistry—including caries detection, early childhood caries risk prediction, and developmental anomaly identification—have achieved promising diagnostic accuracy in controlled research settings, though considerable heterogeneity in imaging protocols and validation designs limits immediate clinical generalizability [82]. For pediatric dental education in China, integrating AI literacy into postgraduate curricula could prepare trainees to critically evaluate and appropriately utilize computer-assisted diagnostic tools, while reinforcing the clinical judgment essential for patient-centered care. However, pediatric-specific validation datasets remain limited, and most AI systems lack robust peer-reviewed assessments of performance in children. Structured faculty training, standardized annotation workflows, and ethical guidelines will be necessary to ensure responsible implementation of these technologies in academic and clinical settings [82].

### 5.4. Reducing the Burden of Disease

The “early detection and management” approach, which focuses on early diagnosis of dental caries and risk assessments, is an effective intervention for preventing the progression of caries [83]. Early diagnosis and risk assessments are crucial in identifying individuals at risk for caries and implementing preventive measures [84].

General practitioners and pediatric dentists play a key role in this process, including providing preventive messaging. However, the low success rate of non-specialists in detecting carious lesions can hinder patient referrals to dentists. To address this, providing training resources such as pamphlets, websites, and online tutorials for non-specialists can improve their ability to detect carious lesions and refer patients to dentists [85]. Multidisciplinary collaboration between dental experts and primary care physicians can also encourage dental referrals from pediatric primary care physicians [58].

Screening children at high risk for caries allows for individualized preventive measures and treatment plans. Caries risk assessment is a critical component of pediatric dentistry, enabling dentists to develop personalized prevention and treatment plans for childhood caries [1]. Risk assessment tools should be utilized to assist dentists in considering treatment options and follow-ups for ECC [86]. However, currently, there is no caries risk assessment model that can accurately predict an individual’s caries development within a specific timeframe. Dentists should utilize existing risk assessment models to tailor effective caries control regimens for their patients.

Additionally, a lack of oral health awareness among parents and caregivers contributes to the high prevalence of pediatric dental diseases. Many parents and caregivers only seek oral health care when their children experience disease-induced pain, rather than focusing on preventive dental care. Approximately 33% of parents or caregivers believe that diseases of deciduous teeth do not require treatment. Passive and active public health interventions, including parental education on dental health knowledge, promotion of oral-related behaviors, increased dental care access, interdisciplinary cooperation, and encouragement of early preventive dental visits, can help prevent common risk factors for oral diseases in children [87]. Oral health and dental camps have become an integral part of educational curricula. School dental health education and the inclusion of oral health information in school textbooks have shown positive impacts on the oral health status, knowledge, and practice behavior of children. Basic and adequate information on oral health included in school textbooks may have beneficial effects on the dental health of school children [88]. Early diagnosis and preventive therapy can reduce the physical and mental burdens, as well as treatment costs for patients, and the financial burden on healthcare institutions. There is a growing emphasis on preventive dental care, including early interventions, fluoride treatments, and education for parents and caregivers to promote good oral hygiene habits from a young age. Furthermore, public health strategies should be developed to increase preventive dental visits and address social disparities in dental care utilization [89,90]. More health workers in local schools, communities, and primary healthcare institutions are needed to enhance the promotion of pediatric dental health knowledge.

## 6. Conclusions

Pediatric dentistry in China faces a range of dilemmas and challenges. Although the government has introduced several oral health-related policies, there are difficulties in effectively implementing them. These challenges include the establishment of grassroots oral healthcare systems, improvement of dental education, and expansion of social insurance coverage. It is crucial to strengthen undergraduate and continuing education in pediatric dentistry and adopt innovative training models. Additionally, the burden of pediatric oral diseases requires attention. Early diagnosis and treatment are essential for preventing the further development of dental caries. However, some individuals without professional training have low ability to recognize dental caries, which hinders them from referring patients to dentists. Therefore, it is necessary to provide training resources to help non-professionals better understand oral health and promote multidisciplinary collaboration between dentists and primary care doctors. Lastly, urban–rural disparities are another aspect that requires attention. Oral health conditions in rural areas of China remain poor, while urban migrant children face limitations due to their families’ economic situations. It is essential to promote rural economic development, leverage dental alliances with universities, enhance local medical teams, raise public awareness of children’s oral health, emphasize preventive care, and establish a referral system between community clinics and specialized dental hospitals.

## Figures and Tables

**Table 1:** Programs adopted by the Chinese government to address children’s oral health issues.

Year of Publication	Program	Purpose
2014	National Children’s Oral Disease Comprehensive Intervention Project Work Specifications [46]	Comprehensively intervene for population oral diseases and promote children’s oral health.
2017	National Nutrition Plan (2017–2030) [47]	Promote nutrition and health, including oral health, by prioritizing the reduction in sugar, oil/fat, and salt intake.
2019	Healthy China Program [48]	By 2030, the prevalence of permanent tooth caries in 12-year-old children should be less than 25%.
2019	Healthy Oral Action Plan 2019–2025 [49]	Improve oral health care for children.
2022	The 14th Five-Year Plan for National Health [50]	Plan and emphasize the enhancement of oral health through the prevention and treatment of common oral diseases such as dental caries and periodontal disease.

**Table 2:** The role and beneficial effects of dental hygienists in different countries.

Country or Region	Implementation of Dental Hygienists	The Role of Dental Hygienists	Beneficial Effects
United States [51,52]	Introduced in 1913 through professional educational institutions.	Lead in coordinating care for early childhood caries; expanded preventive services to address oral health inequities.	Provide early and frequent dental prevention, intervention, and care coordination to reduce oral health disparities.
European Union [53]	In most European countries, dental hygienists practice independently in various settings.	Facilitate dental hygiene services in remote areas, public health services, residential care facilities, and mobile units.	Cost-effective utilization without compromising quality of care; as effective as dentists in diagnosing caries and periodontal diseases.
Islamic Republic of Iran [54]	Integrated into a four-level Dental Health Care System by 1997.	Operate within a four-level system for basic and advanced oral disease management.	Improve oral hygiene, reduce oral disease incidence, decrease dentists’ workload, enhance patient care, promote research and education.
Japan [55]	Recognized as an “exclusive profession” in 1955 and an “exclusive name” in 1989.	Responsibilities include dental prophylaxis, assisting, and health education; perform certain treatments under supervision.	Improved oral health among children and the elderly, reduced caries and periodontal disease incidence, promoted health education.
Korea [56,57]	Dental hygiene process of care introduced in 2005.	Provide interventions based on evidence-based approach, continuity, prevention of oral diseases, and health promotion.	Integration into primary health care system, cooperation with multidisciplinary practitioners to improve general and oral health.

## Data Availability

No new data were created or analyzed in this study. Data sharing is not applicable to this article.

## Abbreviations

The following abbreviations are used in this manuscript:

ECC	Early childhood caries
PBL	Problem-based learning
CBL	Case-based learning
